# Association of adult tobacco use with household food access insecurity: results from Nepal demographic and health survey, 2011

**DOI:** 10.1186/s12889-017-4579-y

**Published:** 2017-07-24

**Authors:** Chandrashekhar T. Sreeramareddy, N. Ramakrishnareddy

**Affiliations:** 1 Department of Community Medicine, International Medical University, Bukit Jalil, 57000 Kuala Lumpur, Malaysia; 2 Department of Community Medicine, Bangalore Medical College and Research Institute, Fort, Bangalore, 560002 India

**Keywords:** Food insecurity, Nepal, Poverty, Tobacco use, Smoking

## Abstract

**Background:**

Food insecurity is a very common problem in developing countries particularly among the poorer households. Very few studies have tested the association between adult smoking and food insecurity.

**Methods:**

We analysed the data from a nationally representative sample of 10,826 households in which women and men (in a sub sample of 4121 households) aged 15-49 years were interviewed in Nepal Demographic and Health Survey 2011. Data from households in which both men and women were interviewed were analysed for association of household food insecurity access score (HFIAS), with tobacco use among men and women, socio-demographic and spatial factors. Univariate comparisons followed by zero-inflated negative binomial regression analyses were done to determine the association between HFIAS and individual, household and spatial factors.

**Results:**

Mean HFIAS score was 3.5 (SD, 4.6) whereas the median was 0 (IQR 0-6). Prevalence of tobacco use among men and women was 50.2% (95% CIs 47.9, 52.6), and 17.3% (95% CIs 15.7, 18.9). HFIAS scores were significantly higher among households where men used tobacco (4.96), and men either smoked or use SLT (3.82) as compared to those without tobacco users (2.79). HFIAS scores were not significantly different by tobacco use status of women. HFIAS score was highest in the poorest households and vice versa. After adjusting for covariates association between HFIAS score and male tobacco use remained significant but effect size decreased when covariates were included into regression models (adjusted OR 1.11). HFIAS score was also associated wealth index (adjusted OR 0.86-0.62) and ecological region (adjusted OR 1.33) and development regions (adjusted OR 1.10-1.21).

**Conclusion:**

Tobacco users in poor(er) households should be encouraged to ‘quit’ their habit. Less affluent sectors of the population also need to be educated about the non-health benefits of quitting, such as improved economic status and reduced food insecurity.

## Background

Food insecurity defined as “‘limited or uncertain availability of nutritionally adequate and safe foods or limited or uncertain ability to acquire acceptable foods in socially acceptable ways” [1] is a well-known determinant of human development [2]. Millions of people in low-and-middle-income countries (LMIC) suffer from extreme hunger and malnutrition [3]; therefore, Sustainable Development Goals (SDGs) have underscored access to food as a basic human right needing urgent measures to end extreme hunger and food insecurity [4]. Food insecurity is linked with poverty [5] and it results in poorer quantity and quality of diet consumed [6]. Nepal is a low-income country where about 40% of its population lives on less than a dollar ($) per day [7]. Household food insecurity(HFI) is widely prevalent in Nepal where 45 of the 75 districts were considered to be food deficient [8] and two-thirds of the households in Kailali district had some degree of food insecurity [9].

Globally, tobacco smoking accounted for about 6.1 million deaths and 143.5 million DALYs in 2013 [10]. Overall smoking rates among men and women are decreasing in high income countries; yet an estimated 967 million smokers live worldwide mainly in LMICs [11]. Both smoking and smokeless tobacco (SLT) use are prevalent in South Asia [12, 13]. Tobacco use not only affects individuals’ health [10, 14] but also affects the household nutrition and budgets especially in low-income houses [15–17]. Families with at least one tobacco user spends up to 20% of the income on buying tobacco products [17, 18] diverting the household income from essential needs such as food, health, education etc. A few studies have demonstrated that adult smoking is associated with food insecurity among the adults and children in the United States of America [19] and with the household food insecurity in Indonesia [20]. Tobacco smoking as well as SLT use are prevalent in Nepal [12]. Since food insecurity, poverty, and tobacco use are all prevalent in Nepal, we further tested, if there was an association between adult tobacco use and household food access insecurity using nationally representative data from Nepal Demographic and Health Survey (NDHS), 2011.

## Methods

### Ethics statement

Ethical approvals for NDHS 2011 and informed consent from the survey participants were obtained as per international guidelines [21]. Before each interview, respondents were informed about details of the survey, voluntary participation, and confidentiality of information and informed consent was obtained. The DHS did not collect any personal information, DHS data were available in the public domain and survey protocols were approved by institutional review boards of the DHS program and the NewERA Nepal, a non-governmental organisation which implemented NDHS, 2011. Therefore, a separate institutional ethical approval was not required to prepare this report.

### Data source

NDHS, 2011 is a population-based, cross-sectional interview study administered by trained personnel. NDHS collected information about households from head of the household who is usually an eldest male member of the family in a patriarchal society of Nepal where joint families are common. At first, households were selected by a two-stage, stratified cluster sampling design, all usual residents of the household were listed and household details were collected on a household questionnaire. This was followed by separate interviews of eligible women (12,674) and men (4121) aged 15-49 years from 10,826 households on separate questionnaires. Since DHS aims to provide reliable data on fertility, family planning, mother and child health, nutrition, etc. they select a large representative sample of women in reproductive age group (15-49 years) but only a sub sample of men are interviewed in the selected households. For this analysis, we included the data collected from men and their wives (corresponding with the women’s data file) living in a sub sample of 4121 households selected for men’s interview in NDHS, 2011. Further details about survey methods of DHS are published elsewhere [22].

### Outcome variable

For the first time, HFI was measured in NDHS, 2011 using the Household Food Insecurity Access Scale (HFIAS) developed by the Food and Nutrition Technical Assistance project. HFIAS measures household’s access to food; one of the three components of food insecurity namely, availability, access and utilization [23]. HFIAS is a low-cost, valid tool applicable in different settings [24] to measure household food insecurity [25]. NDHS, 2011 used a modified HFIAS containing seven of the nine generic questions and the recall period was 12 months instead of 30 days in the original questionnaire. HFIAS was included in household questionnaire administered to the household head. Each question had four response options namely, ‘never’, ‘rarely’, ‘sometimes’ and ‘often’, and were scored as 0, 1, 2 and 3 respectively. Household food insecurity score was the sum of responses to the seven questions could have a minimum of 0 (least food access insecure) to a maximum of 21 (most food access insecure). We used household HFIAS score as an outcome variable in our analyses instead of classifying the households as food insecure or not.

### Tobacco use status variables

In NDHS, 2011 data on tobacco use was collected by the interviewers by asking following four questions to both men and women:

1. *Do you currently smoke cigarettes?* (response as ‘yes’ or ‘no’)
2. *In the last 24 h, how many cigarettes did you smoke?* (response as numerical)
3. *Do you currently smoke or use any other type of tobacco?* (response as ‘yes’ or ‘no’)
4. *What (other) type of tobacco do you currently smoke or use?* (options provided were pipe, chewing tobacco, snuff, and others)

The respondents were classified as ‘current smoker’, if the response to the first question was ‘yes,’ and the response to the fourth question was ‘*bidi*’ and/or pipe (‘*bidi*’ is a type of cigarette made of unprocessed tobacco wrapped in leaves). The respondents were classified as ‘current SLT user’, if their response to fourth question was ‘chewing tobacco’, and/or ‘snuff’. For this analyses, tobacco use was categorised as non-user, smoker/SLT user and dual user. In this analyses, we included any type of tobacco use by men and women; however, we could not quantify tobacco use since daily consumption was collected for cigarette smoking only.

### Covariates

In NDHS, 2011 age of the respondent was collected as the number of completed years. Educational level was classified as ‘no education’, ‘primary’ (up to 4 years of schooling), ‘secondary (five to ten years of schooling),’ or ‘higher’ (college or university and more). Men’s occupation was grouped as ‘unemployed’, ‘professional/services/business’, ‘agriculturist’ and ‘manual worker’. During the household interview given to the household head, all usual members as well as those who slept in the house a day before the survey were counted in the number of household members. Household wealth index was calculated based on a standard set of household assets, dwelling characteristics, and ownership of consumer items which were observed by the interviewer. Based on the household score, each household was ranked by dividing them into quintiles where the first quintile was the poorest 20% of the households and the fifth quintile was the wealthiest 20% [26]. Three spatial variables namely urban or rural area, development region, ecological zone i.e. mountain, hill or plains (known as ‘*terai*’ in Nepali language). For administrative purpose, Nepal is divided into five developmental regions namely Eastern, Central, Western, Mid-western and Far-western.

### Statistical analyses

Descriptive analyses were done for responses to HFIAS questions, HFIAS score, tobacco use and other covariates. At first, an exploratory univariate analyses were done to test the association between HFIAS score and the explanatory variables. Since, the distribution of HFIAS score was skewed, appropriate non-parametric tests were used for comparisons of means (Mann–Whitney *U* test or Kruskal-Wallis Test) and Spearman’s rank correlation tests. Three zero-inflated negative binomial regression models were developed by including tobacco use (in any form) among men and women, demographic variables, household-level and spatial covariates in a step-wise manner to identify the factors associated with HFIAS score. Education was not included in any of the models since we found a strong and significant correlation between men’s educational attainment and wealth index (coefficient + 0.453). We calculated adjusted odds ratios and their 95% confidence intervals by including household weighting factor into the models to account for complex sampling design used in NDHS 2011. A *p*-value 0.05 was considered as significant. All analyses were carried out in Stata version 11.

## Results

The households included for analyses were mainly from rural areas (86.4%); central (24.3%) and eastern (23.7%) regions. Men’s mean age was 29 years (Standard Deviation [SD] 10.2), and women’s mean age was 27.1 years (6.1). Women were mostly not educated (49.8%), whereas men were mostly (52%) educated up to secondary level only, and were either agriculturists (42.9%) or manual workers (31.6%) (Table 1). Among 4121 households selected for men’s survey, HFIAS score was skewed in distribution; mean score was 3.5 (SD, 4.6); median was 0 (interquartile range, 0-6) (Table 2) as HFIAS score in 51% of the households was zero. Three common conditions of HFI reported as occurring *‘often’* during 12 months prior to the survey date were *‘worried about not having enough food’* (18.2%), *‘not able to eat preferred foods because of lack of resources’, (12.8%) and ‘ate limited variety due to lack of resources’* (9.9%) (Table 2). Among men, weighted prevalence rates (%) of smoking, SLT use and any tobacco use were 30.2 (95% CIs 28.1, 32.3), 35.1 (95% CIs 32.7, 37.9), and 50.2 (95% CIs 47.9, 52.6), respectively while the corresponding rates for women were 13.6 (95% CIs 12.7, 14.9), 5.4 (95% CIs 4.5, 6.3), and 17.3 (95% CIs 15.7, 18.9) (data not shown).

**Table 1:** Sample distribution according to socio-demographic factors of the male sub sample in NDHS 2011

Characteristic	Number (%)
Men’s age (mean = 29.0 and SD = 10.2)	-
Women’s age (mean = 27.1 and SD = 6.1)	-
Women’s educational level	
No education	2052 (49.8)
Primary	850 (20.6)
Secondary	1045 (25.4)
Higher	174 (4.2)
Men’s educational level	
No education	498 (12.1)
Primary	815 (19.8)
Secondary	2139 (51.9)
Higher	669 (16.2)
Men’s occupation	
Unemployed	540 (13.1)
Professional/service/business	511 (12.4)
Agriculturist	1303 (31.6)
Manual workers	1767 (42.9)
Household’s wealth index	
Poorest	711 (17.3)
Poorer	688 (16.7)
Middle	729 (17.6)
Richer	861 (20.9)
Richest	1134 (27.5)
Urban/rural area	
Rural	3559 (86.4)
Urban	562 (13.6)
Development region	
Eastern	978 (23.7)
Central	1002 (24.3)
Western	706 (17.1)
Mid-western	781 (19.0)
Far-western	654 (15.9)
Ecological region	
Mountain	618 (15.0)
Hill	1582 (38.4)
Terai (plains)	1921 (46.6)

**Table 2:** The conditions related to food access during the 12 months period in the households included for men’s interview in Nepal Demographic and Health Survey (*n* = 4121)

Question	Never (%)	Rarely (%)	Sometimes (%)	Often (%)
Worried that your household would not have enough food in the past 12 months	56.1	7.8	17.9	18.2
Not able to eat preferred foods because of lack of resources in the past 12 months	58.8	10.2	18.2	12.8
Ate a limited variety due to lack of resources in the past 12 months	60.5	12.4	17.2	9.9
Ate smaller meals because there was not enough food in the past 12 months	79.0	11.0	8.3	1.7
Ate fewer meals in a day because of lack of resources in the past 12 months	85.1	8.5	5.3	1.2
No food to eat because of lack of resources in the past 12 months	86.7	7.8	4.7	0.8
In past 12 months, Went to sleep hungry, because there was not enough food in the past 12 months	92.1	5.2	2.2	0.5
Overall HFIAS score	(mean = 3.5, SD = 4.6) (median = 0, Inter-quartile range 0,6)			

By univariate comparisons of HFIAS scores with dependent variables, HFIAS scores were significantly higher among the households in which men smoked and as well as used SLT(4.96), and men either smoked or used SLT (3.82) as compared to households in which men do not use any form of tobacco (2.79). HFIAS score was also higher among the households where men were agriculturists (4.36) and manual workers (3.62) than those households where men were professionals or in service or business (1.92). HFIAS score showed a negative gradient across men’s educational attainment i.e. highest (6.44) among the households where men were not educated to lowest (1.13) among those with higher education and household wealth index (7.16 in poorest households versus 0.94 in richest households). HFIAS score was also significantly higher among the households in urban areas, Mid-Western and mountainous regions of Nepal. HFIAS scores significantly correlated with number of household members (Table 3). By zero-inflated negative binomial regression analyses, men’s tobacco use was associated with HFIAS score in all three models; however, the effect size slightly decreased in final model when spatial variables were included in the model (adjusted OR 1.18 to 1.11). Women’s tobacco use was not associated with HFIAS score. Development and ecological regions, household wealth index were rather strongly associated with HFIAS score (adjusted OR ratios ranging from 0.57 to 0.87, *p* ≤ 0.001) (Table 4). HFIAS score was not associated with occupation after the inclusion of household and spatial variables into the regression models. However, HFIAS score was not associated with men’s and women’s age, tobacco use by women, sex of household head, and total household members in all models (Table 4).

**Table 3:** Unadjusted associations between household HFIAS score tobacco use and socio-demographic factors by bivariate 14 analyses

	HFIAS score (mean and SD)	test value	*p*-value
Men’s age		O.22	0.894
15-25	3.36 (4.45)		
26-35	3.64 (4.80)		
36-49	3.43 (4.65)		
Women’s age		4.86	0.088
15-25	3.41 (4.55)		
26-35	3.37 (4.55)		
36-49	4.02 (5.03)		
Men’s tobacco use		**39.18**	**<0.001**
None	2.79 (4.08)		
Either smoking or SLT use	3.82 (4.82)		
Dual user	4.96 (5.32)		
Women’s tobacco use		0.773	0.679
None	3.42 (4.56)		
Either smoking or SLT use	3.68 (4.84)		
Dual user	3.33 (4.58)		
Women’s educational level		2.75	0.428
No education	3.35 (4.55)		
Primary	3.51 (4.67)		
secondary	3.55 (4.59)		
Higher	3.79 (4.94)		
Men’s educational level		**556.0**	**<0.001**
No education	6.44 (5.55)		
Primary	5.20 (4.97)		
secondary	2.83 (4.10)		
Higher	1.13 (2.69)		
Men’s occupation		**198.0**	**<0.001**
Unemployed	2.18 (3.77)		
Professional/service/business	1.92 (3.75)		
Agriculturist	4.36 (4.75)		
Manual workers	3.62 (4.75)		
Number of household members^a^		**0.121**	**<0.001**
Sex of the household head			
Male	3.50 (4.66)	0.803	0.337
Female	3.13 (4.15)		
Household’s Wealth Index		**1046.5**	**<0.001**
Poorest	7.16 (5.1)		
Poorer	5.02 (4.64)		
Middle	3.43 (4.29)		
Richer	2.48 (4.07)		
Richest	0.94 (2.48)		
Urban/rural area		**11.8**	**<0.001**
Rural	2.41 (4.18)		
Urban	3.97 (4.71)		
Development region		**184.0**	**<0.001**
Eastern	2.48 (3.92)		
Central	3.11 (4.56)		
Western	3.10 (4.42)		
Mid-western	5.17 (5.19)		
Far-western	3.81 (4.47)		
Ecological zone		**28.17**	**<0.001**
Mountainous	3.77 (4.53)		
Hilly region	3.61 (4.48)		
Tear (Plains)	3.22 (4.71)		

^a^Spearman’s rank correlation test was used, numbers in bold indicate significant associations

**Table 4:** Multivariate linear regression analyses for independent association of HFIAS score with individual, household and spatial explanatory factors

	Model 1		Model 2			
	AOR (95% CI)	*p*-value	AOR (95% CI)	*p*-value	AOR (95% CI)	*p*-value
Any tobacco use by women						
No	Reference		Reference		Reference	
Yes	1.08 (0.99 1.18)	0.083	1.07 (0.99 1.16)	0.094	1.06 (0.98 1.14)	0.169
Women’s age						
15-25	Reference		Reference		Reference	
26-35	0.98 (0.86 1.11)	0.712	0.99 (0.87, 1.12)	0.892	0.99 (0.88 1.13)	0.963
36-49	0.95 (0.83 1.10)	0.508	0.97 (0.84, 1.12)	0.699	0.97 (0.84 1.12)	0.662
Any tobacco use by men						
No	Reference		Reference		Reference	
Yes	1.18 (1.10 1.27)	**<0.001**	1.12 (1.05 1.20)	**0.001**	1.11 (1.04 1.19)	**0.002**
Men’s age						
15-25	Reference		Reference		Reference	
26-35	1.03 (0.96 1.12)	0.405	1.02 (0.95 1.10)	0.592	1.02 (0.95 1.09)	0.628
36-49	1.04 (0.96, 1.13)	0.321	1.04 (0.96 1.13)	0.360	1.03 (0.94 1.12)	0.546
Men’s occupation						
Unemployed	Reference		Reference		Reference	
Professional/service/business	1.05 (0.95 1.17)	0.345	1.05 (0.95 1.16)	0.354	1.03 (0.93 1.13)	0.601
Agriculturist	0.84 (0.73 0.95)	**0.010**	0.89 (0.79 1.01)	0.077	0.91 (0.80 1.03)	0.131
Manual workers	1.01 (0.94, 1.08)	0.845	0.90 (0.84 0.96)	**0.002**	0.94 (0.88 1.00)	0.060
Sex of the household head						
Female			Reference		Reference	
Male			0.97 (0.88 1.06)	0.489	0.96 (0.87 1.06)	0.400
Number of household members			1.01 (0.99 1.02)	0.196	1.01 (1.00 1.02)	0.103
Household wealth Index						
Poorest			Reference		Reference	
Poorer			0.57 (0.52 0.66)	**<0.001**	0.52 (0.45 0.59)	**<0.001**
Middle			0.74 (0.66 0.83)	**<0.001**	0.66 (0.58 0.74)	**<0.001**
Richer			0.80 (0.73 0.88)	**<0.001**	0.74 (0.67 0.83)	**<0.001**
Richest			0.87 (0.80 0.95)	**0.001**	0.84 (0.77 0.93)	**<0.001**
Type of place of residence						
Rural					Reference	
Urban					0.92 (0.83 1.02)	0.108
Development region						
Eastern					Reference	
Central					1.06 (0.96 1.17)	0.216
western					1.18 (1.07 1.31)	**0.001**
Midwestern					1.19 (1.08 1.32)	**0.001**
Far western					1.22 (1.11 1.33)	**<0.001**
Ecological region						
Terai (plains)					Reference	
Hilly region					1.32 (1.17 1.49)	**<0.001**
Mountainous					1.15 (1.04 1.28)	**0.009**

Numbers in bold indicate significant associations

## Discussion

About half the men surveyed were using some type of tobacco product [12] and half the households did not report any food insecurity while the remaining had varying degrees of HFI confirming its existence in Nepal [8, 9]. Our study based on a nationally representative sample has further strengthened association between food insecurity and smoking reported in previous studies [19, 20, 27]. Households in which men used any form of tobacco (smoking and SLT) had highest HFIAS score but association was weaker than association observed with wealth index. Cutler-Triggs et al. studied a large nationally representative sample of 8817 households in the United States of America (USA) reported that living in a house with an adult smoker increased the risk of food insecurity (also its severity) among both children and adults [19]. Another study from the USA reported that smoking was associated with an increased food insecurity in low-income households [27]. Semba et al. studied 26,380 rural households in eight of the 12 provinces in Indonesia found that father’s smoking was associated with increased HFI score [20]. Consistent with previous studies [19, 20], our study also showed that smoking as well as SLT use were associated with higher HFIAS score even after controlling for socio-demographic factors and spatial variables. A study from Indonesia [20] did not asses association between women’s tobacco use and HFI. However, we studied women’s tobacco use which was not associated with HFIAS score. Lack of association may be due to lower tobacco use rates among women, lower quantity of tobacco consumed by women or role of women as mainly housewives in a conservative Nepalese society.

Our findings support the theory that tobacco use diverts household finances away from buying food in developing countries where expenditures on tobacco takes the place of expenditures on food, healthcare etc. particularly in low-income households [15, 17]. Data on household expenditures were not available to explore this association further; nevertheless this seems to be a plausible explanation for association observed between tobacco use and HFI at least in low-income households [15, 17]. Nepalese households spend an estimated 5% of their annual expenditure on tobacco products [28]. However, proportion of all household income spent on tobacco products is more important and this was 22% in poor urban households in Indonesia [29]. Efroymson et al. reported that if limited finances of poor households were not spent on tobacco up to 50% surplus money would be available to buy food or other consumer items [17]. In our study, even after controlling for covariates, wealth index was strongly associated with HFIAS score suggesting that adult tobacco use in the poor(er) households may have deprived them of buying consumer items. Association of tobacco use with socio-economic status is well known in both developing and developed countries [12, 27]. Ironically, in developing countries, tobacco use is significantly higher among economically weaker sections [12]. Kim and Tsoh argue that adults in poorer households may take up smoking to cope with financial stress resulting in a reciprocal effect on food insecurity i.e. poverty increasing smoking which in turn worsens the food insecurity which is a bidirectional association between food insecurity and smoking [30]. It has been hypothesised that smoking may be taken up to overcome psychological distress caused by food insecurity [31] or even to suppress their appetite when food was unavailable [32]. Even after controlling for covariates, HFIAS score was associated with spatial (urban or rural, development regions and ecological zones) factors probably due to residual confounding we could not adjust for in the regression models. Regional disparities in health indicators [22] and food insecurity [33] are well known in Nepal to support our findings.

Our study has following limitations arising from secondary data analyses, design and data collection methods of DHS. Cross-sectional data limits our ability to draw causal inferences of the observed associations in our study. Our results may have unrecognised biases arising from unmeasured explanatory factors not adjusted for in the analyses. Although, effect of poverty on HFI is well known [5], extraneous factors such as ownership of cultivable land, cattle, availability of workforce, rainfall etc. may also affect HFI in rural Nepal, an agrarian country [8]; data on all these factors were not collected in NDHS 2011. Information on daily consumption was not collected for other types of smoking and SLT; hence, we could not include quantity of tobacco consumed into the regression models. Non-validation of modified HFIAS (7-items and 12 months recall period) and extending the period of recall from 30 days to adjust for seasonal variations in food supply in Nepal may have resulted in underestimation of HFI in NDHS 2011. Reporting biases of tobacco use, HFI due to cultural barriers and stigma have been discussed in detail elsewhere [34]. Notwithstanding above limitations, our study further strengthens the reported association of adult tobacco use with HFI, highlighting the previously less recognised non-health consequences of tobacco use on the poor households.

## Conclusion

Tobacco use among economically weaker populations may be a potential hindrance to the success of poverty alleviation and food assistance programs. Tobacco users need to be educated not only about health gains but also financial benefits of quitting tobacco. Tobacco control programs should provide free counselling and medications to help quit tobacco to improve their socio-economic status and food security. In a broader context, the governments should promote food availability and strictly implement tobacco control laws.

## Abbreviations

HFI: Household food insecurity
HFIAS: Household food insecurity scale
LMIC: Low-and-middle-income countries
NDHS: Nepal Demographic and Health Survey
SDG: Sustainable Development Goal
SLT: Smokeless tobacco
